# NucTools: analysis of chromatin feature occupancy profiles from high-throughput sequencing data

**DOI:** 10.1186/s12864-017-3580-2

**Published:** 2017-02-14

**Authors:** Yevhen Vainshtein, Karsten Rippe, Vladimir B. Teif

**Affiliations:** 1 Functional Genomics Group, Fraunhofer Institute for Interfacial Engineering and Biotechnology IGB, Nobelstraße 12, 70569 Stuttgart, Germany; 2 Research Group Genome Organization & Function, German Cancer Research Center (DKFZ) and Bioquant, Im Neuenheimer Feld 280, 69120 Heidelberg, Germany; 3 School of Biological Sciences, University of Essex, Wivenhoe Park, CO4 3SQ Colchester, UK

**Keywords:** MNase-seq, ChIP-seq, Nucleosome positioning, Chromatin, Next-generation sequencing (NGS)

## Abstract

**Background:**

Biomedical applications of high-throughput sequencing methods generate a vast amount of data in which numerous chromatin features are mapped along the genome. The results are frequently analysed by creating binary data sets that link the presence/absence of a given feature to specific genomic loci. However, the nucleosome occupancy or chromatin accessibility landscape is essentially continuous. It is currently a challenge in the field to cope with continuous distributions of deep sequencing chromatin readouts and to integrate the different types of discrete chromatin features to reveal linkages between them.

**Results:**

Here we introduce the NucTools suite of Perl scripts as well as MATLAB- and R-based visualization programs for a nucleosome-centred downstream analysis of deep sequencing data. NucTools accounts for the continuous distribution of nucleosome occupancy. It allows calculations of nucleosome occupancy profiles averaged over several replicates, comparisons of nucleosome occupancy landscapes between different experimental conditions, and the estimation of the changes of integral chromatin properties such as the nucleosome repeat length. Furthermore, NucTools facilitates the annotation of nucleosome occupancy with other chromatin features like binding of transcription factors or architectural proteins, and epigenetic marks like histone modifications or DNA methylation. The applications of NucTools are demonstrated for the comparison of several datasets for nucleosome occupancy in mouse embryonic stem cells (ESCs) and mouse embryonic fibroblasts (MEFs).

**Conclusions:**

The typical workflows of data processing and integrative analysis with NucTools reveal information on the interplay of nucleosome positioning with other features such as for example binding of a transcription factor CTCF, regions with stable and unstable nucleosomes, and domains of large organized chromatin K9me2 modifications (LOCKs). As potential limitations and problems we discuss how inter-replicate variability of MNase-seq experiments can be addressed.

**Electronic supplementary material:**

The online version of this article (doi:10.1186/s12864-017-3580-2) contains supplementary material, which is available to authorized users.

## Background

Numerous chromatin features such as DNA methylation (5mC), histone modifications, binding sites of transcription factors and contact frequencies between enhancers and promoters are linked to gene regulation and transcriptional activity. Many next-generation sequencing (NGS) assays have been developed over the last years to acquire genome-wide maps of these different readouts for analysing chromatin mediated gene regulation. For example, protein binding sites of a given transcription factor (TF) can be determined from chromatin immunoprecipitation with a TF specific antibody followed by sequencing (ChIP-seq) [1–6]. A number of related technologies is applied to determine nucleosome positioning throughout the whole genome [7]. The latter methods usually use either MNase (alone [8–11] or in combination with sonication [12] or exonuclease [13, 14]), or other enzymes such as DNase (DNase-seq) [15, 16], transposase (ATAC-seq) [17, 18] and CpG methyltransferase (NOME-seq) [19]. Another possibility is to use directed chemical cleavage to cut DNA between or inside nucleosomes [20–24]. In addition, nucleosome positions can be mapped by ChIP-seq with antibodies against core histones, e.g. histone H3 [25].

In general, the above NGS methods are based on evaluating small chromatin fragments derived from the genome in terms of a feature of interest and then mapping the resulting sequencing reads to the reference genome. For example, in ChIP-seq experiments, the frequency of chromatin fragments covering each genomic location reflects the abundance of a given feature at a genomic position (e.g. bound protein, or unbound accessible DNA region). Thus, the output of all these methods is a continuous non-homogeneous distribution of sequencing reads along the DNA. Nevertheless, many existing analysis methods treat the results as a discrete distribution of the feature of interest. In practice, this is achieved with the help of peak calling methods. It is assumed that the majority of the signal is just noise that can be disregarded, and only well-defined peaks reflect a biologically relevant chromatin feature. A number of generic computational tools have been developed to perform peak calling, including MACS/MACS2 [26], HOMER [27], SICER [28], PeakSeq [29] and CisGenome [30] to name just a few. Furthermore, there are many specialised programs that perform peak calling to determine nucleosome positions [7], including TemplateFilter [10], NPC [31], nucleR [32], NOrMAL [33], PING/PING2 [34, 35], MLM [36], NucDe [37], NucleoFinder [38], ChIPseqR [39], NSeq [40], NucPosSimulator [41], NucHunter [42], iNPS [43] and PuFFIN [44]. However, the binary classification of genomic positions into occupied or free is not always justified. In many cases the underlying biology is such that the feature distribution along the DNA cannot be treated as discrete. This is particularly relevant for nonspecific or weakly specific protein binding, as well as the nucleosome distribution along the DNA. In these cases it is more appropriate to operate with continuous occupancy profiles to identify regions with cell type/state specific differential occupancy. A straightforward approach to define regions of differential occupancy is to shift a sliding window along the genome and count the number of reads at each window position. This has been implemented, for example, in the DANPOS/DANPOS2 [45], DiNuP [46] and NUCwave [47] software packages. Continuous genomic maps resulting from this type of analysis frequently need to be associated with discrete genomic features like promoters, enhancers, etc. Thus, the downstream workflow is different than the one used for binary chromatin feature maps.

Here we introduce the NucTools software package, which provides computational protocols for a nucleosome-centred NGS downstream analysis. As input our framework uses raw DNA reads from BAM/SAM files mapped with programs such as Bowtie/Bowtie2 [48, 49], NGM [50] or BWA [51], which are then converted into the BED format for further processing. Basic manipulations with BED files can be performed using the popular BEDTools package [52]. BEDTools conducts most basic operations like dataset intersection, format conversion and enrichment analysis. Similar to this concept, our NucTools software package provides flexible solutions for most typical nucleosome-centred analyses. Several excellent user-friendly “all-in-one” packages for ChIP-seq data analysis like Crunch [53], ChAsE [54], CAGT [55], CisGenome [30] and deepTools [56] already exist. However, these lack nucleosome-specific functions or customization options to process billions of nucleosome reads in a parallelized manner. NucTools, on the other hand, provides a modular framework devoted primarily to nucleosome positioning. It is composed of several independent open-source scripts, each solving a particular task, which can be combined or extended in a highly scalable workflow, typically detailed using bash files on a Linux cluster. The framework contains several functions specific for nucleosomes. However, it can be also used for similar types of NGS analysis beyond nucleosome positioning. It is particularly useful for the integration of datasets with a continuous chromatin feature density distribution. In the following section we will first outline the basic concepts and provide the overview of a typical NucTools workflow. Subsequently, the application of NucTools to several recent nucleosome positioning datasets in mouse embryonic stem cells (ESCs) and mouse embryonic fibroblasts (MEFs) is demonstrated.

## Implementation

**Fig. 1:**
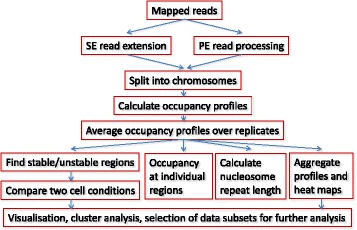
An exemplary analysis workflow using NucTools. BAM/SAM files with raw mapped reads are converted to BED format (bowtie2bed.pl), processed to obtain nucleosome-sized reads (extend_SE_reads.pl or extend_PE_reads.pl), and split into chromosomes (extract_chr_bed.pl). Usually, a separate directory with chromosome bed files is created for each sample similarly to the HOMER’s approach. Afterwards chromosome-wide occupancies are calculated and averaged using a window size suitable for the following analysis (bed2occupancy_average.pl). Then for each cell type/state, an average profile is calculated based on the individual replicate profiles (average_replicates.pl). After this point several types of analysis can be performed in parallel: Finding stable/unstable regions (stable_nucs_replicates.pl); comparing replicate-averaged profiles in different cell states/types (compare_two_conditions.pl); calculating nucleosome occupancy profiles at individual regions identified based on the intersection of stable/unstable regions or regions with differential occupancy with genomic features such as promoters, enhancers, etc. (extract_rows_occup.pl); calculating the nucleosome repeat length (nucleosome_repeat_length.pl and plotNRL.R); calculating aggregate profiles or visualizing heat maps of nucleosome occupancy at different genomic features (Cluster Maps Builder). The next types of analysis usually involve gene ontology, multiple-dataset correlations and DNA sequence motif analysis, which can be conducted for the genomic regions of interest identified at the previous steps using external software packages

Sequencing data processing usually starts with mapping DNA reads with tools such as Bowtie/Bowtie2 [48, 49], NGM [50] or BWA [51]. In the discrete binding site-type analysis, subsequent steps to identify the localization of a chromatin feature of interest involve peak calling with programs like MACS/MACS2 [26], HOMER [27], SICER [28], PeakSeq [29], edgeR [57] and CisGenome [30]. Unlike discrete binding site analysis, NucTools is based on the concept of continuous occupancy distribution and includes also regions of low read density. This type of analysis makes use of the complete data set and evaluates properly averaged quantities to characterize chromatin features under different cell conditions. A typical NucTools workflow is represented Fig. 1.

Our pipeline starts with preparatory steps such as read pre-processing to convert short mapped DNA reads to nucleosome-size DNA fragments (or, dependent on the type of experimental input data, dinucleosomes or larger complexes). In the case of single-end sequencing experiments one has to extend the reads in a strand-specific manner with the estimated average fragment length to obtain bed file with coordinates of both ends of each sequenced DNA fragment. In the case of paired-end sequencing, reads are usually stored as two consecutive lines in .bed files. It is convenient to convert them into one line, which contains the start and the end of the DNA fragment. These steps are achieved by our scripts extend_SE_reads.pl and extend_PE_reads.pl for single-end and paired-end reads correspondingly. In the case of single-end reads, the exact length of the nucleosome fragment is not known and needs to be provided by the user as a parameter. This parameter can be either determined experimentally (e.g. using Agilent Bioanalyzer) or estimated by NucTools with the help of the script calc_fragment_length.pl provided in the package.

The next preparatory step is splitting reads into separate files per chromosome. This step might not seem obvious, since in the case of discrete data such as TF binding sites or histone modifications it is more convenient to keep all the peaks together in one bed file. This is technically feasible without problems since a typical number of regions in these cases is limited to tens of thousands sites with typical file sizes of several megabytes. However, in the case of continuous analysis for nucleosome positioning, we are dealing with billions of reads and file sizes of order of several gigabytes, which becomes relevant for computer memory allocation for the subsequent analysis steps. Therefore, NucTools splits reads into chromosome-wide files that are obtained with the help of the script extract_chr_bed.pl. Note that a similar approach of splitting files into chromosomes is also employed by HOMER [27]. All chromosomes are usually stored in the same directory so that the directory name can be used as an input parameter instead of file names of individual chromosome files. In order to save storage space, our scripts can generate gzipped output and take gzipped files as input.

In the next step BED files with mapped reads are converted to chromosome-wide nucleosome occupancy files. Our occupancy files have the default extension .occ and contain two columns: the genomic coordinate and the signal value (e.g. nucleosome occupancy) for a given coordinate. Calculating the occupancy with single base pair resolution results in a file size for one human chromosome of ~1-2 Gb. To accelerate calculations and decrease storage and memory requirements, our script bed2occupancy_average.pl allows a user to select a window size, and report average values for each genomic window of a given size, e.g., a window of 100 bp will make files 100 times smaller. We recommend keeping these files during the whole following analysis rather than recalculating them. This saves computational time at the expense of the storage space and is particularly useful for large-scale projects.

At the heart of our method is the averaging and normalisation of the data using several replicate experiments. The nucleosome positioning analysis for human or higher eukaryotes requires billions of reads and several replicates for the same experimental condition in order to be robustly interpretable [58]. We call these datasets “replicates” for generality, while in practice some of these data can be from unrelated laboratories, which use different experimental protocols for the same cell state/type as demonstrated below. For each replicate, the strength of the MNase-seq or ChIP-seq signal critically depends on the quality of antibody, chromatin digestion conditions, sequencing depth and variations of the experimental protocol [59–63]. Therefore, cross-platform comparison of datasets obtained in different laboratories is challenging [64–66]. Several solutions to normalise datasets have been proposed in the literature, such as ChIPnorm [67], ChIP-Rx [68], NCIS [69], MACE [70] and CisGenome [30]. The normalization strategy depends on the biological question. For example for TF ChIP-seq, one approach is to do peak calling, determine common peaks which are represented in all replicates, and then normalize the datasets such that the common peaks on average retain the same heights [71]. In contrast, for nucleosome positioning we normalize each replicate to its sequencing depth with a sliding window of a user-defined size (e.g. 100 bp, etc.). The normalized occupancy O_N_ is calculated as O_N_ = <O_R_> / (nuc_size * N_R_ / chr_length). The parameter < O_R_ > is the average occupancy in the given window, nuc_size is the average size of the nucleosome fragment, N_R_ is the number of reads in the input BED file, and chr_length is the length of the chromosome excluding unmappable regions at the chromosome ends, which is calculated by the script.

At the next step one can determine stable/unstable nucleosome occupancy regions for a single cell state. The relative error of defining nucleosome occupancy using different replicates can be used as a proxy to determine stable versus unstable (“fuzzy”) nucleosomes. This is achieved with the script stable_nucs_replicates.pl. This script allows a user to select a threshold value for the nucleosome occupancy and the relative error – the threshold value depends on the type of analysis which needs to be conducted. For example, it can be used to find different classes of nucleosome occupancy regions, such as DNA linkers free from nucleosomes or regions with moderately or extremely stable nucleosomes, or regions with labile nucleosomes/high nucleosome turnover. A user has to select the sliding window size and which signal is used for the filtering (e.g. occupancy or fuzziness). As output this script returns the list of genomic regions in a modified BED file format. This file contains the chromosome, region start and region end columns followed by the columns quantifying the average signal value for a given window (usually the nucleosome occupancy), and the absolute and relative error based on the replicate comparison. The relative error is calculated as the ratio of the standard error based on all replicates to the value of the average signal.

Another type of analysis with NucTools is finding genomic regions which have changed their nucleosome occupancy between different cell conditions, e.g. during cell differentiation or between tumor cells and controls from healthy donors. From the genomic locations of stable and unstable nucleosomes identified at the previous step regions that change nucleosome occupancy or stability can be determined. This analysis is conducted with the script compare_two_conditions.pl to determine ensemble-average differences of the nucleosome occupancy or stability between two cell states. By selecting the appropriate column as the signal, a user can choose whether the comparison is conducted for the nucleosome occupancy for identifying regions of gained/lost nucleosomes, or for the relative error to identify regions that are more/less fuzzy in terms of nucleosome positioning. The user can define a threshold value for the differences in occupancy or relative error between two cell conditions, and thus make the nucleosome subset larger/smaller. Alternatively, the resolution of the analysis for differential nucleosome occupancy can be determined by the window size. Obviously, these parameters are dependent on the type of the downstream analysis and the biological question. In the example below we will consider two extreme cases of different biological analyses: megabase-size regions and nucleosome-size regions. Once the subset of genomic regions with lost/gained or fuzzy/stable nucleosome has been defined with compare_two_conditions.pl, it can be further analysed using motif discovery tools, such as HOMER [27], MEME [72], Weeder, Pscan and PscanChIP [73], rVISTA [74] and other programs. Another possible direction of downstream analysis for such a subset of genomic location is an annotation with Gene Ontology (GO) terms using several existing online tools, such as DAVID [75], GOrilla [76], EnrichR [77] and GREAT [78].

Another typical application of our analysis workflow is extracting chromatin maps from multiple datasets for individual genomic regions. While genome browsers such as the UCSC Genome Browser [79] or IGV [80] are very convenient to look at different tracks on individual genomic regions, their snapshots are often not optimal for the quantitative analysis. On many occasions we had to manually assemble a figure, where several smoothed curves representing different chromatin signals were plotted together and normalized to the same scale (different TFs, nucleosome positioning, etc.). To make this kind of plots one has to extract from the occupancy file a subset of rows within a given genomic interval. This is achieved by script extract_rows_occup.pl. The visualization can then be performed with plotting software of choice as for example Origin (originlab.com) or the visualization tools available in R. A more sophisticated use of the region extraction script is testing a certain hypothesis using statistical methods for many user-defined regions. An example of this kind of analysis is the comparison of predicted and experimentally observed transcription factor binding occupancies [81], as e.g. in the case of the interplay of CTCF binding and nucleosome positioning in our previous work [71]. In such cases the script extract_rows_occup.pl can be called in a cycle for all regions of interest.

Another analysis step, which is usually missing in existing software packages, is the calculation of the nucleosome repeat length (NRL). This type of analysis is specific to nucleosome positioning and is conducted with the script nucleosome_repeat_length.pl. It evaluates the average distance between the centres of neighbouring nucleosomes. The script takes as input the raw mapped reads and calculates the frequency of distances from the leftmost end of a given nucleosome read and leftmost ends of all nucleosome reads in its vicinity, typically within the region of 1000–3000 bp (parameter --delta determined by the user). The resulting distribution of frequencies of start-to-start nucleosome distances has peaks at distances between nucleosomes separated by 0, 1, 2, 3, 4 or more linkers. The algorithm used in this calculation was initially described by Valouev et al. [82] and updated in our following publications [83, 84]. The distribution of nucleosome start-to-start distances determined by nucleosome_repeat_length.pl can be the analysed by an R script plotNRL.R, which extracts peak coordinates and performs linear fitting; the slope of the line gives the NRL [83]. NRLs can be compared either between different regions of the same cell, or between different cell states for the same genomic regions. For example, the NRL in the regions around CTCF is about 10 bp smaller than genome average [83, 84], while NRL changes during cell differentiation can be as large as dozens of base pairs [82, 85–87].

Further downstream analysis steps typically link nucleosome occupancy maps to other datasets such as gene expression, DNA methylation or histone modifications [83, 84]. These analyses usually aim to answer questions such as whether the sequencing signal in dataset A is correlated with feature B, or with signal from dataset C as well as more complex logical conditions. There are many computational tools that can address some of these questions, but there is no single tool that can solve all of them, since these questions are quite diverse. It is not uncommon that software tools for this step are developed specifically for a given project [88–90]. One possibility to find correlations between different datasets is to calculate pair-wise correlation functions using all the data including the noise, as is done with the MCORE software [91]. Another possibility is to calculate the colocalization of different datasets for certain genomic features (binding sites, etc.). NucTools focuses on the latter option implemented in the script aggregate_profile.pl. This script allows the calculation of the coverage maps for many genomic regions aligned with respect to some common feature. Individual coverage maps can be visualized in a heat map using our standalone MATLAB-based program Cluster Maps Builder (CMB). This program is included in the NucTools distribution as MATLAB source files as well as precompiled executable files for Windows operating system so that it may be run without requiring a MATLAB licence (see details on the NucTools web site). The ordering of the regions can be performed according to several clustering algorithms selected by the user. We recommend using k-means clustering for a typical nucleosome analysis. Alternative clustering programs of similar kind are GAGT [55] and deepTools [56]. An important feature of the CMB is that it allows performing clustering for one experimental condition, and then saving it and applying exactly the same clustering order to another experimental condition. Note that such an analysis requires prior resorting and matching of all involved datasets: the number of features and the original sorting order in each dataset should be the same. The corresponding R script (match_2tables_byID.R) is included in our package. Cluster Maps Builder allows dissecting clusters of genomic regions which are characterized by a similar profile of ChIP-seq (MNase-seq, etc) density, then extracting the regions from these profiles and performing further downstream analysis. After each clustering run all generated figures are saved automatically and the IDs of all genomic regions and corresponding occupancy profiles can be saved separately for each cluster. These IDs can be then conveniently converted to a BED file with genomic coordinates using a script merge2tabs.pl provided in NucTools, allowing further downstream analysis. One example of such analysis could be to predict differential TF binding from biophysical models, and compare continuous profiles predicted by the theory with the experimental ChIP-seq data [71]. Another task addressed by script aggregate_profile.pl is the integration of ChIP-seq and DNA methylation data. The problem is that most existing software packages only deal with the coordinates of differentially methylated regions for this purpose (an approach analogous to peak calling). On the other hand, it may be useful to take advantage of the single base pair resolution of DNA methylation data as obtained by bisulfite sequencing. DNA methylation positions obtained from standard methylation callers such as Bismark [92] can be converted into occupancy files with the continuous DNA methylation coverage in analogy to ChIP-seq using bed2occupancy_average.pl, thus making these datasets directly comparable. Then the script aggregate_profile.pl provides a possibility to deal with all individual methylated or unmethylated cytosines (a user can define the threshold level of individual cytosine methylation). For example, it is possible to calculate cluster maps or aggregate profiles aligning all nucleosomes around >20 millions of CpGs in the mouse genome, as was done in our previous works [71], and *vice versa* one can calculate the density of DNA methylation around any genomic feature [71].

## Results and discussion

In the next section we demonstrate the application of NucTools to mouse embryonic stem cell (ESC) differentiation. ESCs represent a very well-defined cell line used for chromatin analysis in many laboratories. Several hundred high-throughput sequencing datasets exist for this cell type [93]. Importantly, more than 14 datasets of nucleosome positioning in ESCs determined by MNase-seq listed in a recent review [7] have been reported by about 10 different laboratories including ours [71, 84]. Nucleosome positions derived from these datasets overlap only partially. Thus, identifying stably bound nucleosomes with a peak-calling type of analysis is fraught with difficulties. Here we demonstrate how NucTools can be applied to analyse nucleosome occupancy in ESCs in comparison to mouse embryonic fibroblasts (MEFs) as their differentiated counterparts. The MNase-seq data sets for ESCs from Voong et al. [24] (“complete digestion”, GSM2183911), West et al. [94] (two replicates, GSE59062) and Zhang et al. [95] (two replicates, GSE51766) are used and compared to two MNase-seq datasets in MEFs from our previous publication [84] (GSM1004654).

**Fig. 2:**
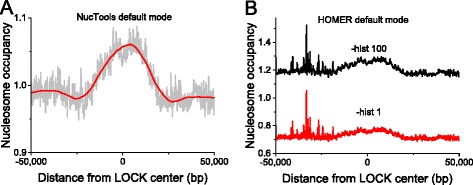
Aggregate profiles showing nucleosome density around the centres of LOCK regions (large organized chromatin K9me2 modifications) in ESCs [96]. **a** Calculation using NucTools (*grey*) and the corresponding Savitzky-Golay smoothing of this curve (*red*). A clear increase of nucleosome density is seen as a characteristic of LOCKs. **b** Calculation using HOMER in its default mode. Large peaks resulting from sequencing artefacts seen on the left from the centre preclude proper identification of the shape of the aggregate profile. HOMER’s advanced mode -histNorm allows suppressing these artefacts making the curve more similar to the curve in panel (**a**) (data not shown). The accumulation of sequencing artefacts strongly interfering with large-scale analysis of aggregate profiles is a standard problem

Figure 2 shows the results of the calculation of the aggregate nucleosome occupancy profile based on the MNase-seq data from Voong et al. [24] around the centers of so-called LOCK. The latter represent large histone H3 lysine 9 dimethylated chromatin blocks [96], which have been previously mapped in ESCs using H3K9me2 ChIP-seq. Our calculation using NucTools shown in Fig. 2a suggests that LOCK are characterized by a higher than average nucleosome density, which is in line with the paradigm that they are similar in their function to heterochromatin regions. LOCK regions have large sizes (~50 kb), and there are relatively few of them (*N* = 2,559). Due to these peculiarities the calculation of the same aggregate profile using HOMER in its default mode is less effective (Fig. 2b). The profile calculated by HOMER still allows one to guess the curve shape similar to the one calculated by NucTools in panel 2a, but it is less clear due to artefacts on the left side of the plot. HOMER has also an advanced mode “-histNorm” where such artefacts can be suppressed, after which the curve becomes less noisy and more similar to the one calculated by NucTools (data not shown). The artefact suppression is realized differently in NucTools and HOMER. HOMER removes sequencing artefacts by disregarding low-occupancy regions, while NucTools removes artefacts by disregarding regions with suspiciously high occupancy. In our experience, the latter filtering works somewhat better. This artefact filtering is hard-wired in our script aggregate_profile.pl. The user usually does not need to adjust it but four other different normalization options are available for advanced users as detailed in the program’s manual. On the other hand, the size of the region to be taken into account in the calculation is obviously an analysis-specific parameter which needs to be selected by the user. Here, we selected a region [−50,000, 50,000], which is determined by the LOCK region sizes.

**Fig. 3:**
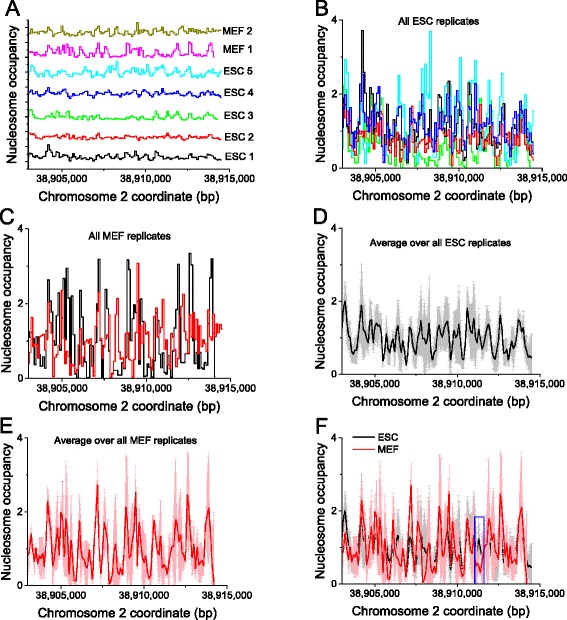
Different representation of nucleosome occupancy profiles at an individual genomic region (promoter of gene Golga1). 100-bp window averaging was performed using script bed2occupancy_average.pl for five experiments in ESCs reported by Voong et al. [24] (denoted ESC 1), West et al. [94] (denoted ESC 2 and ESC 3) and Zhang et al. [95] (denoted ESC 4 and ESC 5) and two experiments in MEFs from our previous publication [84] denoted MEF 1 and MEF 2. **a** A genome browser-style representation of all nucleosome occupancy tracks. **b** All ESC tracks superimposed. **c** All MEF tracks superimposed. **d**, **e** The average profiles calculated correspondingly over all ESC and all MEF experiments using script average_replicates.pl. The *grey and light red* areas show the standard deviation. **f** The averaged ESC and MEF profiles are superimposed on the same figure. An exemplary genomic region where the difference between the two profiles is significant is indicated by the *blue rectangle*

Figure 3 demonstrates different views of multiple nucleosome positioning tracks for a single genomic region that can be obtained with NucTools. The representation in panel 3a is typical for genome browsers – several signal tracks stacked on top of each other. Such a representation is useful when looking at features which have well-defined peaks, but is suboptimal in the case of the continuous noisy nucleosome occupancy landscapes. In this particular case, it is very difficult to spot any significant differences between the five ESC replicates and two MEF replicates shown on the figure. One problem is that the lines need to be plotted together rather than on top of each other in order to be quantitatively comparable. However, even if plotted together as in panels 3b and 3c, we can only see that the replicate experiments significantly differ, but still cannot make any quantitative conclusions. These panels demonstrate the general problem in the field that quantification of nucleosome occupancy profile requires many replicates and large amount of sequencing in mammalian cells for good statistics. Importantly, there is usually no “consensus” nucleosome profile, because each replicate experiment reflects slightly different experimental conditions. With NucTools, we can determine which regions in the nucleosome landscape are relatively stable across all replicate experiments, and which regions are more variable. This is accomplished with the script average_replicates.pl. As a result, an average profile is obtained for ESCs (panel 3d) and for MEFs (panel 3e). The comparison of the two average profiles reveals the differences between ESCs and MEFs (panel 3f). In this particular case, we can identify a region where nucleosome occupancy changes significantly between ESCs and MEFs (shown by the blue rectangle in panel 3f).

**Fig. 4:**
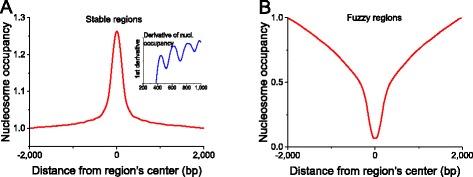
Aggregate profiles showing different properties of the nucleosome occupancy signatures at stable and fuzzy 100-bp genomic regions calculated using stable_nucs_replicates.pl for the data from GSM2183911 (complete MNase-digestion of wild-type ESCs [24]). **a** Stable regions have increased nucleosome occupancy and act as a boundary statistically positioning nearby nucleosomes. The insert shows regular oscillations of the 1^st^ derivative of the nucleosome occupancy. **b** Fuzzy regions have decreased nucleosome occupancy and are not associated with specifically positioned nucleosomes. These are preferentially nucleosome-depleted regions such as active promoters and enhancers

As another example, NucTools is applied to the genome-wide analysis of nucleosome occupancy. Firstly we have determined genomic regions which contain stable and unstable nucleosomes in ESCs using script stable_nucs_replicates.pl. A sliding window of 100 bp was used and stable regions were selected as those where the relative error based on five ESC replicates <0.2, while this value was set to >2 for unstable (“fuzzy”) regions. With these parameters 1,193,318 stable and 376,850 unstable regions are obtained. Next the aggregate nucleosome occupancy profiles around the centers of these regions were calculated. Figure 4a shows that that the stable regions defined above are characterized by increased nucleosome occupancy. Furthermore, one can spot slight oscillations of the nucleosome occupancy adjacent to the main peak. To better visualize these small oscillations the first derivative of the nucleosome occupancy is plotted in the insert. The peak of nucleosome occupancy at the center of stable regions together with the oscillations of nucleosome occupancy at adjacent regions suggests that regions of this class contain strongly positioned nucleosomes. These may act as statistical barriers for creating regular nucleosome arrays in their vicinity. Further analysis of this dataset using EnrichR [77] supports this idea by linking these regions to H3K9me3 histone modification characteristic for stable nucleosome arrays [84]. On the other hand, the aggregate profile of nucleosome occupancy around unstable (“fuzzy”) regions is characterized by significant nucleosome depletion. It is noted that our definition of stable and unstable nucleosomes was independent of the occupancy value. Rather, the characteristic chromatin density increase and decrease correspondingly for stable and unstable regions was obtained as a result of filtering genomic regions by the level of the relative error based on the five ESC replicates. The regions that show variable nucleosome occupancy between replicates are preferentially nucleosome depleted. Unlike stable regions, in this case the curve of the aggregate nucleosome occupancy is very smooth and does not reveal oscillations. Thus, regular nucleosome arrays are preferentially associated with stable and not unstable regions.

At the next analysis step the differences in nucleosome occupancy between ESCs and MEFs were evaluated. The end user of NucTools can define these differences in a number of ways depending on the type of the following downstream analysis and the biological question of interest. As an example the differences between stable nucleosome regions as defined above in ESCs versus MEFs are computed. The script compare_two_conditions.pl takes as input results of the script stable_nuc_replicates.pl, and reports differences based on the user-selected signal and threshold, e.g. either comparing the occupancy in ESCs and MEFs, or comparing the fuzziness in ESCs and MEFs. Here, we selected nucleosome occupancy as the signal and the threshold of the relative occupancy change as 0.99. The relative occupancy change O_diff_ is calculated by the script as O_diff_ = 2 * (<O_N1_ > − < O_N2_>) / (<O_N1_ > + < O_N2_>), where < O_N1_ > is the replicate-averaged occupancy in a given genomic region in the experimental condition 1, and < O_N2_ > is the replicate-averaged occupancy in the experimental condition 2. A total of 21,205 100-bp regions were obtained where nucleosome occupancy increased in MEF versus ESCs, and in 200,909 100-bp regions nucleosome occupancy decreased in MEF versus ESCs. In our experience the asymmetry between the numbers of regions which gained and lost nucleosomes is quite systematic and probably reflects biological differences between the cell states. EnrichR analysis of these datasets reveals that the regions which gain and lost nucleosomes in MEFs versus ESCs are associated with two distinct sets of transcription factor binding motifs listed in Additional file 1: Table S1 and Additional file 2: Table S2 (TBP, SRF, CBEBP, Sox2, IRF2, GATA1, JUND, POU2F1, CPEB1 in the case of gained nucleosomes, and TFAP2A, SP1, NFKB1, TEAD2, RELA, KLF13, NR1I2, CRX, MYC, IKZF1 in the case of lost nucleosomes). This distinction may indicate different mechanisms of nucleosome loss and gain during ESC differentiation.

**Fig. 5:**
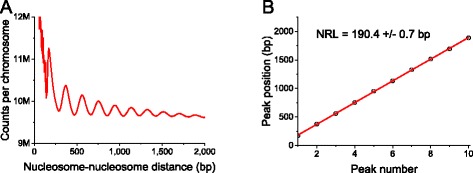
Calculation of the NRL for ESCs based on the data from GSM2183911 (complete MNase-digestion of wild-type ESCs [24]) using scripts nucleosome_repeat_length.pl and plotNRL.R. **a** The average frequency of nucleosome-nucleosome distances genome-wide. **b** Peak positions plotted as a function of the peak numbers from panel (**a**). The linear fit of these points reveals the NRL and the error of its determination. In this case, NRL = 190.4 ± 0.7 bp. This is the genome-average NRL. NRLs calculated for smaller genomic regions may differ from each other; the genome-wide NRL is the average of all local NRLs

Figure 5 shows the results of NucTools calculation of the nucleosome repeat length in ESCs based on the dataset from Voong et al. [24] (“complete digestion”, GSM2183911). In this case, NRL = 190.4 +/− 0.7 bp. Interestingly, our previous estimation of the nucleosome repeat length in ESCs was about 4 bp smaller. This reflects the intrinsic variability of this type of experiments. While it is safe to compare NRLs between different genomic regions based on a single experiment, for the comparison of different cell states a very rigorous statistics needs to be performed using several different replicates as exemplified in Fig. 3.

**Fig. 6:**
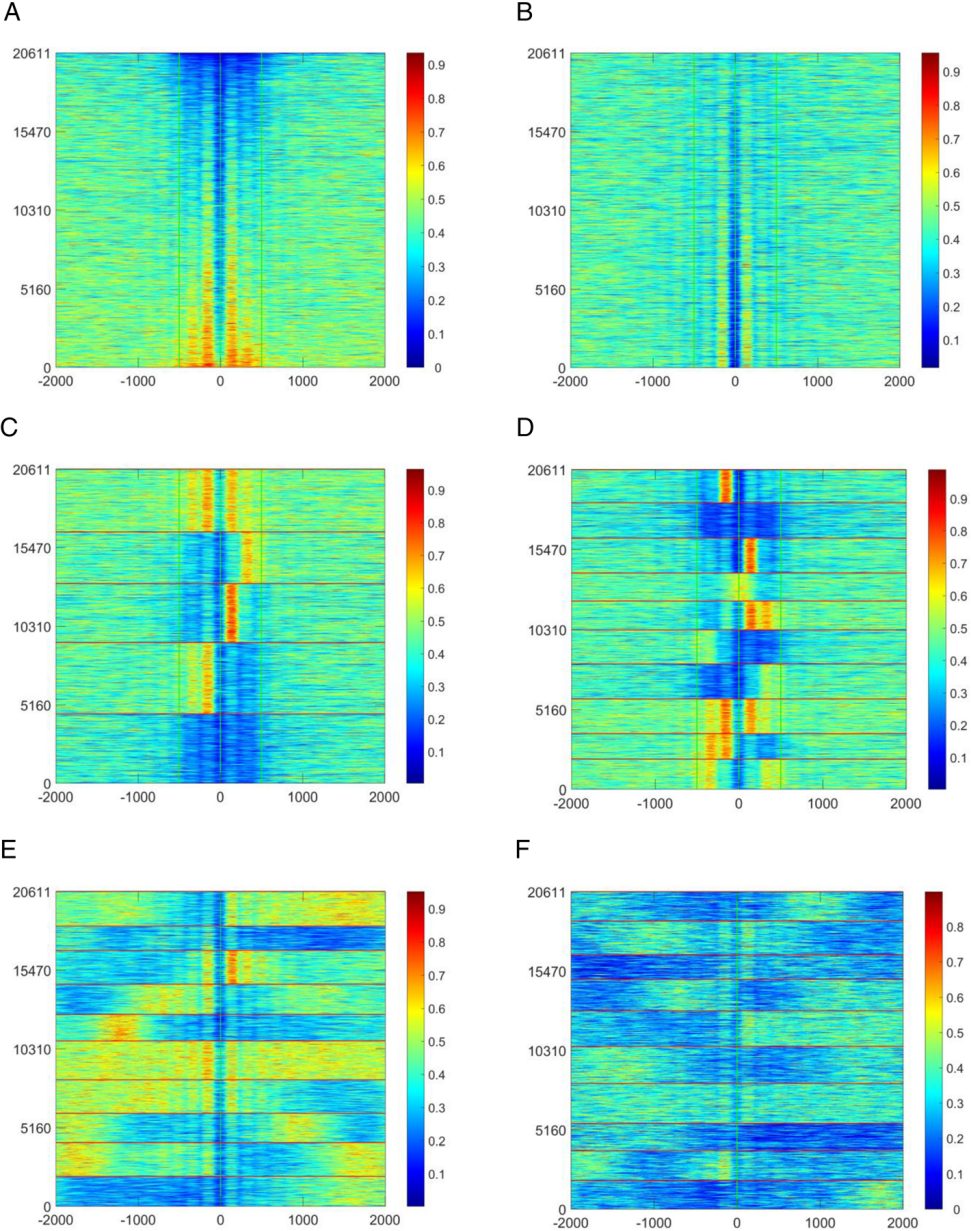
Exemplary heat maps calculated using Cluster Maps Builder. **a**–**e** Nucleosome occupancy in ESCs from Voong et al. [24] (“complete digestion”, GSM2183911) around common CTCF sites present both in ESCs and MEFs defined as in [84], sorted according to the average occupancy value in the [−2000, 2000] region (**a**), CTCF binding site score (**b**), k-means clustering with 5 clusters based on nucleosome occupancy in the [−500, 500] region (**c**), k-means clustering with 10 clusters based on nucleosome occupancy in [−500, 500] region (**d**), k-means clustering with 10 clusters based on nucleosome occupancy in [−2000, 2000] region (**e**). **f** Nucleosome occupancy in MEFs [84] (GSM1004654) around common CTCF sites present both in ESCs and MEFs, sorted as in panel **e**

Figure 6 shows the heatmaps calculated using the NucTols’ Cluster Maps Builder program for the nucleosome occupancy in ESCs and MEFs around common CTCF sites which are present both in ESCs and MEFs defined as in [84]. The nucleosome occupancy oscillation around bound CTCF is a well-known feature [71, 83, 84, 97]. Figure 6a shows the heatmap calculated for the nucleosome occupancy in ESCs determined by Voong et al. [24] (“complete MNase digestion”, GSM2183911) around common CTCF sites, with the sorting order determined by the average value of nucleosome occupancy in the region [−500, 500] around CTCF site. Figure 6b re-orders the same data following the CTCF binding site score from smallest CTCF ChIP-seq peaks (top) to the largest CTCF peaks (bottom). Interestingly, the larger the CTCF peak, the more pronounced is the nucleosome depletion. This is consistent with the classical hypothesis of nucleosome/CTCF competition and argues against the nucleosome occupancy peak centered at CTCF-bound sites based on the chemical mapping data reported in the same publication by Voong et al. [24]. (One possible explanation could be that the chemical nucleosome mapping which works by introducing an artificial cysteine in the middle of the nucleosome might interfere with a similar signal from natural cysteines that are part of CTCF). Figure 6c reorders the same data by performing k-means clustering for 5 clusters based on the nucleosome occupancy in the region [−500, 500] around CTCF. One can see that different subsets of CTCF-bound sites are actually characterised by different nucleosome signatures – a similar conclusion was reached earlier by Kundaje and coauthors [55]. Figure 6d reorders the same data using k-means clustering for 10 clusters based on the nucleosome occupancy in the region [−500; 500]. Figure 6e also uses k-meand clustering for 10 clusters, but now a larger region [−2000, 2000] is taken into account when calculating the similarities between nucleosome occupancy patterns. As a result, the latter type of analysis allows visualizing nucleosome occupancy oscillations extending to the whole region shown in the heat map. Finally, Fig. 6f keeps the same region order as in Fig. 6e, but reports the calculations performed for the nucleosome from one of the replicates of MNase-seq in MEFs [84]. The comparison between Fig. 6e and f reflects not only the biological changes between ESCs and MEFs, but also a difference between the sequencing depths in ESCs (~1 billion reads) and MEFs (~150 million reads). As a result the fine features of the nucleosome occupancy distribution are better distinguishable in ESCs. Importantly, NucTools allows conveniently extracting all subsets identified using cluster analysis in Fig. 6 for further downstream analysis of the corresponding genomic regions.

## Conclusions

Here, we have introduced the software package NucTools for a continuous chromatin feature analysis. Typical workflows and the application to a specific example of nucleosome repositioning and occupancy changes during differentiation of ESC differentiation were illustrated. The NucTools set of scripts addresses the need to cope with the continuous distribution of genomic nucleosome occupancies and multiple large datasets and provides an approach to integrate other chromatin features complementing already available third party computational tools. Some of the problems described above like inter-replicate variability are not just technical but rather conceptual. Thus, there is an ongoing need to address these issues with additional theoretical approaches and we will extend and update the NucTools as these become available.

## Availability and requirements

**Project name:** NucTools

**Project home page:** https://homeveg.github.io/nuctools

**Archived version:** http://www.generegulation.info/index.php/nuctools

**Operating system(s):** Platform independent for core scripts; Windows 7 for CMBT

**Programming languages:** Perl, R, MatLab

**License:** GNU GPL 3 or higher

**Any restrictions to use by non-academics:** None

## Additional files

Additional file 1: Table S1. EnrichR analysis of the enrichment of DNA sequence motifs based on TRANSFAC and JASPAR PWMs in 100-bp genomic regions which gained nucleosomes in MEFs. (PDF 29 kb)
Additional file 2: Table S2. EnrichR analysis of the enrichment of DNA sequence motifs based on TRANSFAC and JASPAR PWMs in 100-bp genomic regions which lost nucleosomes in MEFs. (PDF 29 kb)
