# Supplementary material for: NucTools: analysis of chromatin feature occupancy profiles from high-throughput sequencing data
Source: BMC Genomics. 2017 Feb 14;18:158. doi: 10.1186/s12864-017-3580-2 (PMC5309995; doi:10.1186/s12864-017-3580-2)
Supplement: Additional file 1: Table S1. — EnrichR analysis of the enrichment of DNA sequence motifs based on TRANSFAC and JASPAR PWMs in 100-bp genomic regions which gained nucleosomes in MEFs. (PDF 29 kb) [file 12864_2017_3580_MOESM1_ESM.pdf]

Table S1. EnrichR analysis of the enrichment of DNA sequence motifs based on TRANSFAC and JASPAR PWMs in 100-bp genomic regions which gained nucleosomes in MEFs.

Index	Name	P-value	Adjusted p-value	Z-score	Combined score
1	TBP	1.363e-22	4.266e-20	-1.55	68.97
2	SRF	1.967e-15	3.078e-13	-1.64	47.22
3	CBEPB	1.093e-11	1.141e-9	-1.57	32.25
4	Sox2	2.425e-10	1.898e-8	-1.66	29.56
5	IRF2	1.603e-9	1.003e-7	-1.61	25.98
6	Gata1	6.211e-9	3.240e-7	-1.59	23.83
7	JUND	8.788e-9	3.929e-7	-1.61	23.75
8	POU2F1	4.666e-8	0.000001825	-1.61	21.22
9	CPEB1	2.152e-7	0.000006735	-1.63	19.39
10	NFYB	2.152e-7	0.000006735	-1.62	19.24
