# Supplementary material for: NucTools: analysis of chromatin feature occupancy profiles from high-throughput sequencing data
Source: BMC Genomics. 2017 Feb 14;18:158. doi: 10.1186/s12864-017-3580-2 (PMC5309995; doi:10.1186/s12864-017-3580-2)
Supplement: Additional file 2: Table S2. — EnrichR analysis of the enrichment of DNA sequence motifs based on TRANSFAC and JASPAR PWMs in 100-bp genomic regions which lost nucleosomes in MEFs. (PDF 29 kb) [file 12864_2017_3580_MOESM2_ESM.pdf]

Table S2. EnrichR analysis of the enrichment of DNA sequence motifs based on TRANSFAC and JASPAR PWMs in 100-bp genomic regions which lost nucleosomes in MEFs.

Index	Name	P-value	Adjusted p-value	Z-score	Combined score
1	TFAP2A	4.028e-15	1.261e-12	-1.69	46.18
2	SP1	3.287e-14	5.144e-12	-1.63	42.29
3	NFKB1	4.129e-11	4.308e-9	-1.52	29.23
4	TEAD2	1.881e-9	1.472e-7	-1.70	26.68
5	RELA	1.060e-8	6.633e-7	-1.60	22.80
6	KLF13	7.026e-8	0.000003099	-1.66	21.11
7	NR1I2	1.269e-7	0.000003316	-1.65	20.84
8	CRX	1.141e-7	0.000003316	-1.65	20.77
9	MYC	8.365e-8	0.000003099	-1.59	20.13
10	IKZF1	7.717e-8	0.000003099	-1.58	20.08
